# How Consumption May Be Prevented

**Published:** 1883-01

**Authors:** 


### HOW CONSUMPTION MAY BE PREVENTED.

Professor Tyndall sends to the London *Times* a letter giving the summary of a lecture recently delivered in Berlin by Dr. Koch showing the results of his researches to prove that tubercular consumption is caused by a parasite. In giving an account of Koch's experiments, he says:

"Of six guinea pigs, all in good health, four were inoculated with bacilli derived originally from a human lung, which in fifty-four days had produced five successive generations. Two of the six animals were not infected. In every one of the infected cases the guinea pig sickened and lost flesh. After thirty-two days one of them died, and after thirty-five days the remaining five were killed and examined. In the guinea pig that died, and in the three remaining infected ones, strongly pronounced tubercular disease had set in. Spleen, liver and lungs were found filled with tubercles, while in the two uninfected animals no trace of the disease was observed. In a second experiment, six out of eight guinea pigs were inoculated with cultivated bacilli, derived originally from the tuberculous lung of a monkey, bred and rebred for ninety-five days, until eight generations had been produced. Every one of these animals was attacked, while the two uninfected guinea pigs remained perfectly healthy. Similar experiments were made with cats, rabbits, mice and other animals, and without exception it was fouud that the injection of the parasite into the animal system was followed by decided and, in most cases, virulent tubercular disease.

"In the cases thus far mentioned inoculation had been effected in the abdomen. The place of inoculation was afterward changed to the aqueous humor of the eye. Three rabbits received each a speck of bacillus culture, derived originally from a human lung affected with pneumonia. Eighty-nine days had been devoted to the culture of the organism. The infected rabbits rapidly lost flesh, and after twenty-five days were killed and examined. The lungs of every one of them were found charged with tubercles. Of three other rabbits, one received an injection of pure blood-serum in the aqueous humor of the eye, while the other two were infected in a similar way with the same serum containing bacilli derived originally from a diseased lung and subjected to ninety-one days' cultivation. After twenty-eight days the rabbits were killed. The one which had received an injection of pure serum was found perfectly healthy, while the lungs of the two others were found overspread with tubercles.

"Other experiments are recorded in this admirable essay, from which the weightiest practical conclusions may be drawn. Koch determines the limits of temperature between which the tubercle-bacillus can develop and multiply. The minimum temperature he finds to be eighty-six degrees Fahrenheit, and the maximum one hundred and eighty-four degrees. He concludes that, unlike bacillus anthracis of splenic fever, which can flourish freely outside the animal body, in the temperate zone animal warmth is necessary for the propagation of the newly discovered organism. In a vast number of cases Koch has examined the matter expectorated from the lungs of persons affected with phthisis and found in it swarms of bacilli, while in matter expectorated from the lungs of persons not thus afflicted he has never found the organism. The expectorated matter in the former case was highly infective, nor did drying destroy its virulence. Guinea pigs infected with expectorated matter which had been kept dry for two, four and eight weeks respectively, were smitten with tubercular disease quite as virulent as that produced by fresh expectoration. Koch points to the grave danger of inhaling air in which particles of the dried sputa of consumptive patients mingles with dust of other kinds."

The London *Medical News* says of the possible results of this discovery:

"If Pasteur's culture experiments have led to the discovery of a method by which the poison of splenic fever is rendered harmless, and the disease prevented by the timely inoculation of the modified virus, may we not hope that the time is not distant when the ravages of consumption will be prevented by the inoculation of a modified bacillus? The medical profession of the whole civilized world will now await with the keenest interest the developments which may be expected from further study of the bacillus tuberculosis."

